# Health care professionals’ views of the factors influencing the decision to refer patients to a stroke rehabilitation trial

**DOI:** 10.1186/s13063-015-1115-1

**Published:** 2015-12-18

**Authors:** Nessa Thomas, Sarah Plant, Kate Woodward-Nutt, Yeliz Prior, Sarah Tyson

**Affiliations:** Salford Royal NHS Foundation Trust, Salford, UK; School of Nursing, Midwifery & Social Work, University of Manchester, Manchester, UK; Centre for Health Sciences Research, University of Salford, Salford, UK; Mid Cheshire Hospitals NHS Foundation Trust, Cheshire, UK; Stroke Research Centre, University of Manchester, Jean McFarlane Building, Oxford Road, Manchester, M13 9PL UK

**Keywords:** Recruitment, Decision-making, Selection bias, Rehabilitation trials, Stroke

## Abstract

**Background:**

Effective recruitment is an essential element of successful research but notoriously difficult to achieve. This article examines health care professionals’ views on the factors influencing decision-making regarding referral to a stroke rehabilitation trial.

**Methods:**

Semi-structured interviews and a card-sorting task were undertaken with stroke service staff in acute and community hospital trusts. Data analysis used a thematic framework approach.

**Results:**

Twenty-seven qualified health care professionals from 12 (6 acute and 6 community) hospital trusts and one charity participated. Four main factors emerged: patient-related, professional views, the organisation and research logistics, which all contributed to staff’s decision about whether to refer patients to a trial.

Clinicians identified patient-related factors as the most frequent influence and considered themselves the patients’ advocate. They used their knowledge of the patient to anticipate the patients’ reaction to possible participation and tended to only refer those whom they perceived would respond positively.

Participants also identified experience of research, a sense of ownership of the project and a positive view of the intervention being evaluated as factors influencing referral. The need to prioritise clinical matters, meet managerial demands and cope with constant change were organisational factors impacting negatively on referral. Staff often simply forgot about recruitment in the face of other higher priorities. Quick, simple, flexible research processes that were closely aligned with existing ways of working were felt to facilitate recruitment.

**Conclusions:**

Patient- and professional-related factors were the most frequent influence on clinicians’ recruitment decisions, which often had a ‘gate-keeping’ effect. Managerial and clinical responsibility to juggle multiple (often higher) priorities was also an important factor.

To facilitate recruitment, researchers need to develop strategies to approach potential participants as directly as possible to enable them to make their own decisions about participation; ensure that research processes are as quick and simple as possible; align with existing clinical pathways and systems; and give regular reminders and ongoing support to promote recruitment.

**Trial registration:**

ISRCTN, 98287938. Registered 6 May 2015

**Electronic supplementary material:**

The online version of this article (doi:10.1186/s13063-015-1115-1) contains supplementary material, which is available to authorized users.

## Background

Effective recruitment of participants is an essential element of successful research but notoriously difficult to achieve [1]. Difficulty reaching recruitment targets are well established and many potential solutions have been suggested; however, best practice is still unclear [2–5]. Typically, recruitment to a research study involves a clinician or research support staff (usually a doctor or nurse) screening health care records to identify patients meeting the selection criteria. Eligible patients are then approached by a clinician or research support staff who explains the study to them and ascertains their interest in participation. Thus, these health care professionals’ decisions about whether to approach patients and an understanding of the factors which influence such decisions are key to facilitating effective recruitment. This has received relatively little attention, as much work has focussed on promoting recruitment, or the understanding of research processes rather than the factors behind decision-making.

A recent meta-synthesis of health care professionals’ perceptions of recruitment processes selected 18 papers working in 10 clinical specialties (most frequently oncology) and primarily involving doctors, nurses, midwives and pharmacists. They identified five factors influencing recruitment decisions: the relationship between researcher and clinicians; resources available; nature of the research and its acceptability to clinicians and patients; impact on professional identities and recruitment strategies [6]. Contemporaneously, an in-depth interview study of doctors and nurses involved in recruitment revealed that many felt a conflict between their research role to promote recruitment and their role as clinicians. However, they were largely unaware of the impact this had on recruitment, instead focussing on organisational and patient-related barriers [7].

We aimed to explore the factors which influence clinicians’ decisions about whether to refer to a research study to identify issues that could facilitate recruitment. As stroke rehabilitation researchers, we particularly wanted to involve allied health professionals and those working in community- and outpatient-based stroke rehabilitation services who are rarely included in studies of research methods and processes.

## Methods

This was a mixed-methods qualitative study, which used semi-structured interviews and a card-sorting exercise (detailed below) to elucidate participants’ views. The study was undertaken as an embedded study within a stroke rehabilitation trial: the Ankle Foot Orthosis for Stroke (AFOOT) trial—ISRCTN 98287938, registered May 2015. Ethical approval was obtained from University of Manchester’s Ethics Committee and the Lancaster Committee of National Research Ethics Service (reference 11/NW/0325).

AFOOT is a feasibility trial to compare two commonly used types of ankle foot orthosis (AFO). These are used by stroke survivors to support their weak foot and ankle and prevent their toes from catching or their ankle turning over while walking. We recruited community-dwelling stroke survivors who had limited mobility (but could walk short distances without assistance of another person) and had not previously been issued with an AFO. We stratified stroke severity and whether they were receiving post-hospital rehabilitation. There were no limits in the time since stroke or age. Once consent was obtained, participants completed baseline assessments and were fitted with either an off-the-shelf or a custom-made AFO by their local orthotics department. Outcome (patient satisfaction with the AFO; gait speed; functional mobility; falls risk and adverse events) assessments were made 6 and 12 weeks post-baseline.

We aimed to recruit 140 participants, which was achieved to time and target after an unfunded extension to accommodate the impact of a major structural re-organisation of health care services. An important aspect of the trial feasibility was to identify effective recruitment strategies. Our target population (community-dwelling stroke survivors with limited mobility) have generally been discharged from stroke services. Thus, past and present patients of community-based therapy and rehabilitation services were our main ‘recruitment sources’, which are managed separately from stroke and primary care services. In the UK, these services are not included in research support infrastructure, such as the clinical research networks, and so the AFOOT research team had to build new collaborative relationships and provide support and training to build research capacity and capability amongst these clinicians who had little, or no, previous involvement in research. To develop this support, both for this group and more generally, we wanted to understand the factors which influenced their decision-making about whether to approach a patient about taking part in the trial.

To recruit participants in this study, we contacted all the health care professionals who had been directly involved in making decisions about referral for the AFOOT trial during the latter stages of its recruitment period, by personal contact, email and newsletters. Staff with an academic or research role in their job were excluded. Purposive sampling ensured a range of professions, staff grades and experience of stroke care and research were represented. Recruitment continued until saturation was judged by the research team to be reached. Those who responded to say they were interested in taking part were given a participant information sheet and opportunity to ask any questions. Then, the face-to-face interviews were arranged at their convenience. At the beginning of the interview, informed consent was taken.

Data collection meetings lasted approximately 1 h. Firstly, a semi-structured interview was undertaken, which was audio-recorded, transcribed verbatim and anonymised. An interview schedule (see Additional file 1) was developed taking themes from the literature, the authors’ discussions with clinicians involved recruitment to the research group’s studies and our broader experience of recruitment to research studies. The schedule was piloted with two stroke research therapists. Minor changes were made to the wording of questions and the probes/prompts used. The authors undertaking the interviews (NT, KWN, SP) also met regularly to deal with any problems and agree any iterations of the interview schedule.

The card-sorting task was then completed to ensure that some specific issues would be addressed by all participants, to provide objective information, to address issues which could not be covered in the interviews in the time available and to triangulate the interview data. Statements regarding issues which may, or may not, influence HCPs’ decisions to refer a patient to a trial were produced from the pilot interviews, the literature and the authors’ experience. They were piloted within the research team and three independent stroke therapists. Any which were ambiguous, felt to be clearly irrelevant or duplicated other statements were removed. A final collection of 60 statements were selected. We expected there to be some redundancy in the collection (i.e. some statements which would not be chosen) as we felt it was important to capture issues which were not thought important as well as those that were.

After the interview was complete, participants were given the statement cards. They were asked to identify statements which were likely to influence their decision (either positively or negatively). The order in which the cards were presented was re-randomised for each participant.

A framework analysis approach [8] was used to analyse the interview data using NVivo10 software for data coding, cross-referencing, storage and retrieval. This involved the authors reading and re-reading the first 15 transcripts to familiarise themselves with the data and making a list of the initial themes and sub-themes identified. Then, line-by-line open coding of each transcript was undertaken independently by at least two of the authors to establish the interconnectedness of the themes to develop a framework. This was collectively refined and charted against demographic information and annotated field notes to maximise its grounding in the data. This process was repeated for the other interviews while they were ongoing to inform further development of the interview schedule (if needed) and ensure data saturation had been reached.

Data from the card-sorting exercise were analysed descriptively and using geometric coding. This is a novel method of descriptive analysis that enables combinations of chosen statements to be identified [9]. Each statement was given a unique code from numbers in the sequence *a*(*n*) *=* 2^*n*^ (1,2,4,8,16,32, . . .). This is a geometric progression that is a sum-free sequence which means each number is never a sum of the preceding numbers. Therefore, when the numbers are summated, there is only one combination of numbers (or statements) that can produce that summated number (or geometric code). Thus, the combinations of statements that give the geometric code can be identified.

## Results

Twenty-seven participants took part in the study (Table 1). Twenty were physiotherapists, two were occupational therapists and two were orthotists. A rehabilitation ward matron, prescribing advanced practitioner and a stroke physician also participated. On average, they had been qualified for 13 (SD 9) years. Fourteen (52 %) were employed by an acute hospital, 12 by a community-based hospital or service and one by a charity. Previous research experience was mixed. Three (11 %) had acted as a principal investigator; 16 (59 %) had been involved in referring patients to participate in the AFOOT trial and/or other research; 14 had been involved in recruitment (52 %); 9 (33 %) had recruited a patient to a case study or undertaken their own recruitment as part of post-graduate study. All had been identified as potential referrers to the AFOOT trial.

**Table 1:** Details of the participants

Profession	Grade	Gender	Years since qualification
Physiotherapist	Junior (NHS Band 5)	Male	8
Physiotherapist	Junior (NHS Band 5)	Female	3
Physiotherapist	Junior (NHS Band 5)	Female	4
Occupational therapist	Specialist (NHS Band 6)	Female	10
Physiotherapist	Specialist (NHS Band 6)	Female	8
Physiotherapist	Specialist (NHS Band 6)	Female	5
Physiotherapist	Specialist (NHS Band 6)	Female	5
Physiotherapist	Specialist (NHS Band 6)	Male	9
Physiotherapist	Specialist (NHS Band 6)	Female	9
Physiotherapist	Specialist (NHS Band 6)	Male	4
Physiotherapist	Specialist (NHS Band 6)	Female	7
Nurse	Senior specialist (NHS Band 7)	Female	11
Occupational therapist	Senior specialist (NHS Band 7)	Female	23
Physiotherapist	Senior specialist (NHS Band 7)	Female	9
Physiotherapist	Senior specialist (NHS Band 7)	Female	9
Physiotherapist	Senior specialist (NHS Band 7)	Female	14
Physiotherapist	Senior specialist (NHS Band 7)	Female	10
Physiotherapist	Senior specialist (NHS Band 7)	Female	14
Physiotherapist	Senior specialist (NHS Band 7)	Female	24
Physiotherapist	Senior specialist (NHS Band 7)	Female	30
Nurse	Consultant/manager (NHS Band 8)	Female	25
Physiotherapist	Consultant/manager (NHS Band 8)	Female	23
Physiotherapist	Consultant/manager (NHS Band 8a)	Female	30+
Physiotherapist	Consultant/manager (NHS Band 8a)	Female	20
Orthotist	Consultant/manager (NHS Band 8)	Male	23
Orthotist	Consultant/manager (NHS Band 8)	Male	30
Specialist stroke physician		Male	4

Twenty-four participants completed the card-sorting exercise. One participant undertook the card-sorting exercise but felt none of the statements were relevant; their decision about whether to refer to a trial was based on the eligibility criteria alone. The two orthotists did not take part in the card-sorting as they had had no prior knowledge of the patients and therefore could refer on known eligibility criteria only.

Four overarching themes emerged from the interviews and the card-sorting exercise which concerned the patient, the professional, the organisation and the research. The statements in the card-sorting exercise were categorised to reflect these themes.

The number of statements chosen ranged from 3 to 28 (mean = 12, SD 7, median = 5). The most frequently chosen statement (#1: ‘I want my Trust to be recognised for Research & Development activities’) was chosen 17 times. Eleven statements (#49–#60) were never chosen; these mostly related to research knowledge. The statements, their identifying number and how often they were chosen are detailed in Table 2. The most frequently chosen statements related to the patient (*n* = 153); the professional (*n* = 64); the organisation (*n* = 39) and research logistics (*n* = 22).

**Table 2:** The statements, themes to which they belong and the number of times they were chosen

Statement number	Theme	Sub-theme	Statement	Frequency chosen
1	Organisation	Organisation	I want my Trust to be recognised for Research & Development activities	17
2	Patient-related	+ve attribute	Patient likes to try new things	14
3	Patient-related	+ve attribute	Patient is very motivated	13
4	Patient-related	−ve attribute	Patient tends not to comply and is overwhelmed by too many things going on at once	11
5	Patient-related	Treatment	Patient is doing well increasing mobility with current treatment plan	11
6	Patient-related	−ve attribute	Patient refuses to do most things	10
7	Patient-related	−ve attribute	Patient is overwhelmed	10
8	Patient-related	Stroke-related	Patient is receptively dysphasic	9
9	Patient-related	Stroke-related	Patient would never manage to get an AFO on	9
10	Patient-related	Stroke-related	Patient is fatigued and has to prioritise what they can and can’t do	8
11	Patient-related	Uncertainty	Unsure how the patient will react to uncertainty	8
12	Individual	Professional	If we had been involved in the development of the project I would be keener to refer	8
13	Patient-related	Family	Family tend to be protective and tend not to be keen on new ideas	7
14	Patient-related	Treatment	Referring is likely to alter our therapeutic relationship (either positively or negatively)	7
15	Research logistics	AFOOT support	Route to contact Research Team is too slow	7
16	Individual	Professional	We are always having students asking about these types of projects	7
17	Patient-related	−ve attribute	Patient finds it difficult to stick to things	6
18	Patient-related	Treatment	Ready to Discharge Patient	6
19	Organisation	Service provision	The Study follow-up is longer than our standard treatment window	6
20	Individual	Professional	Don’t know patient well (e.g. covering colleagues caseload)	6
21	Patient-related	Stroke-related	Patient is expressively dysphasic	5
22	Patient-related	Uncertainty	Unsure how the patient will react	5
23	Patient-related	+ve attribute	Patient is well read up on stroke	5
24	Organisation	Organisation	Unsure of Manager’s views of me prioritising research	5
25	Organisation	Service provision	Difficult to cover current caseload, I see research as an extra	5
26	Individual	Research knowledge	Don’t have enough details to be certain the patients were appropriate	5
27	Organisation	Service provision	It is too much work to refer with everything else that’s going on	5
28	Individual	Professional	It was unlikely that the patient would benefit from either splint	5
29	Patient-related	Stroke-related	Patient had visual deficits which made reading the information difficult	4
30	Organisation	Service provision	Time is pressured. There is time for, either, extra treatment *or* research referral	4
31	Research logistics	Support	Route to contact Research Team is too time consuming	4
32	Research logistics	Support	Research Team can’t keep pace with treatment plans	4
33	Individual	Professional	Previous poor experience with Orthotics	4
34	Individual	Professional	There is other research I would prefer to put patients forward for	4
35	Research logistics	Support	I suggested patients during handover but never heard anything more about it	4
36	Patient-related	Patient	Patient doesn’t have any sensible shoes	3
37	Individual	Professional	There are more ideal patients	3
38	Research logistics	AFOOT support	Unsure of referral process	3
39	Individual	Professional	I don’t think that the research question is important	3
40	Individual	Research knowledge	Only the more Senior grades can refer	3
41	Organisation	Service provision	The Trial seemed complex, there wasn’t time to find out more details	3
42	Patient-related	−ve attribute	Patient sees the negative in everything	2
43	Individual	Research knowledge	Previous poor experience of research	2
44	Individual	Research knowledge	It’s their treating physio’s decision, leave it to them	2
45	Individual	Research knowledge	Don’t feel confident to talk to patients about research	2
46	Individual	Research knowledge	It’s a team decision, there isn’t time to get everybody’s agreement	2
47	Individual	Professional	There are posters up advertising the trial, I would prefer patients to self-refer	1
48	Individual	Professional	There is too much research, it’s confusing who is doing what	1
49	Individual	Research knowledge	Don’t agree patients should be approached whilst they are trying to recover	0
50	Individual	Research knowledge	NHS don’t pay me to do this extra work	0
51	Individual	Research knowledge	Previous experience of complications for patients involved in research	0
52	Individual	Research knowledge	Don’t feel confident to talk to patients about AFOs	0
53	Individual	Research knowledge	We have a member of the stroke team who takes care of all research	0
54	Individual	Research knowledge	Referral to research is not my decision to make	0
55	Individual	Professional	Haven’t had a patient who has had any success with an AFO	0
56	Individual	Research knowledge	My professional insurance doesn’t cover me for research	0
57	Individual	Professional	There are more important research questions so I wouldn’t prioritise this one	0
58	Individual	Research knowledge	Can’t refer because of patient confidentiality	0
59	Organisation	Organisation	There’s nothing in it for my Trust, it only benefits the University	0
60	Individual	Professional	We already know about these AFOs so no need to research further	0

No combination of statements was chosen more than once. All but one participant (*n* = 23) chose at least one statement relating to the patient; and only two did not choose any professional-related statements (*n* = 22). Three did not choose any statements relating to the organisation (*n* = 21), and 12 (*n* = 12) did not choose a research logistic-related statement.

### Patient-related factors

Patient-related factors most frequently influenced decisions about whether to refer a patient. The most frequently chosen statements related to:

- Patients’ personal attributes:
  - positively in that participants were more likely to refer if patients were motivated and/or willing to try new things (statements #2 and #3)
  - negatively; referral was less likely if patients appeared non-compliant; overwhelmed by their stroke or refused to do most things (#4–#6)
- Stroke-related factors such as uncertainty whether the patients fitted the inclusion criteria (#8, #18, #23, #26) or whether post-stroke fatigue would make taking part in the trial too taxing for the patient (#10)
- Whether the intervention on offer would be feasible (#9)
- The family’s perceived lack of support for their relative to be involved in research (#13)
- Uncertainty: uncertainty about how patients would react made participants less likely to refer patients (#11)
- The potential impact of participation on the patient’s current treatment plan (#5 and #18)
- Concerns that asking patients to participate may influence the therapeutic relationship between professional and patient either positively or negatively (#14)

This revealed a high degree of ‘gate-keeping’. If participants were uncertain about whether the patient met the inclusion criteria, would want to be involved or would benefit from it, they would make a decision on their behalf rather than giving the patient an opportunity to make the decision themselves. In the interviews, some participants detailed how they used their own values and preferences to make decisions on the participants’ behalf: ‘I try and think, if it was me and I’d been admitted and I suddenly couldn’t move… and then someone asked me about research, it’s just quite a lot to process isn’t it’ (Interviewee I515).

The broader impact of a trial intervention, particularly the emotional aspects, were frequently raised as an influence on the decision to refer: ‘You’d maybe be a bit wary about directing someone towards something, if you knew they might get very into the idea but then it had come to a stop at the end of the trial; the emotional side of that*…*.’ (Interviewee I516). The impact that participating in a research study may have on patients’ expectation and the current treatment plan were also a factor as Interviewee I510 explained: ‘Those that we’re not working with in depth ….. we don’t think about approaching something new. [We] do our little episode and leave it at that really, rather than open up something more’*.*

Participants did not only act as patients’ advocates, a degree of cherry picking was evident as many discussed looking for ‘good’ patients to refer: ‘They just have to be ticking the entry box, but not this perfect patient that …. would be good to interview, and got a nice house for you to go and see. You almost sort of can talk yourself out of somebody being suitable I think. So, there’s that little bit to it, I think I want a nice patient…. We don’t want somebody that’s just going to DNA [not attend] you all the time’ (Interviewee I510).

### Professional views

Most participants recognised that their individual views influenced decisions about referral for a research study. In the card-sorting exercise, all but two participants chose at least one statement relating to their professional views. The frequently chosen individual-related statements related to their:

- Involvement in developing the research study (#12)
- Previous experience, knowledge and confidence about research (#16 and #26)
- How well they knew the patient (#20)
- View of the treatment (#28 and #33)
- Value attached to the research topic, design and demands of other (preferred) ongoing research studies (#34)

Most of the less frequently chosen statements related to participants’ knowledge and confidence about research (statements #40, #43–#48). In the interviews, several participants described how their opinion (both positive and negative) of a trial intervention and how it fitted with their usual practice would influence their decision to refer a patient. However, in contrast, others identified how involvement in the AFOOT trial had made them more aware of the possible benefits of the trial intervention and therefore to refer patients. More often participants had strong views and preferences about the way the trial invention was delivered and were less likely to refer if these were not reflected in the trial. Others did not refer to the inclusion criteria when considering referral but based decisions on their personal view and established practice.

### The organisation: service demands and ethos

This theme fell into three categories: the ethos, juggling priorities and the impact on the service, and constant change.

#### Organisational ethos

Although not prominent in the interviews, the card-sorting exercise revealed that participants most frequently chosen factor influencing participants’ decision-making was their employers’ organisational ethos (#1). The other organisational factor that figured in participants’ recruitment decisions was the support (or otherwise) of their line manager (#24).

#### Juggling priorities and the effect on the service

Other frequently chosen statements related to the impact of involvement in research (both the referral process and the trial intervention) on the service (#19, #25, #27, #30). In the interviews, participants explained these issues in more detail. For example, in the AFOOT trial, there was a mismatch between the timescales for usual care (physiotherapy) and the trial intervention (fitting an ankle foot orthosis by the orthotics department) as most orthotics departments had waiting lists of several months. This would extend the timescale of the physiotherapist’s treatment period and have a negative effect on their service activity statistics: ‘I [physiotherapist] can only see patients for between 6–8 weeks…I don’t want to have to refer them to wait for an orthotist for weeks and weeks.’ (Interviewee I508).

The influence of other clinical and professional demands on the priority given to recruiting patients was frequently raised. Most felt research was an optional extra (#19) which was necessarily prioritised below other demands, particularly their clinical workload.

The organisational demands (such as work that would contribute to achievement of service quality targets) were also influential as Interviewee I516 explained: ‘Just at the minute, particularly, we have so much additional stuff to try and remember to do with patients. We do lots of patient feedback surveys, loads of patient related outcome measures, things like that. There’s just a lot of paperwork and things to remember to go through.’

#### Constant change

The study was undertaken during a period of health service re-organisation. Many participants felt they were working in an unstable environment. The impact on staffing was particularly severe. Many participants described high staff turnover from ‘rotating staff’ (junior posts which rotate clinical area every few months to gain a broad experience); flexible working policies; an extended working week (6- or 7-day working) and locum staff to cover high vacancy rates. This put pressure on permanent staff to ensure information and training about ‘active’ research studies was cascaded. Information giving and training needed to be repeated frequently to new staff (as there were few occasions when staff were altogether) and permanent staff found it difficult to keep track of who knew what. Multi-disciplinary team meetings were identified as an efficient way to deliver this information, but not all staff attended them while others would repeatedly hear the same information.

Some staff queried whether it was reasonable to expect junior staff to assimilate research information and ‘get up to speed’ with recruitment processes during a short rotation (typically 4 months). As Interviewee I516 explained: ‘Our Band 5 [junior grade] physio rotates every four months. So it’s quite a quick turnover really, and Band 6 s rotate every six months. There’s constant new people coming through the team. With all the other stuff we have to throw at them in the induction, it’s not always, I guess, a top priority’.

### Research logistics

The final theme regarded the logistics of complying with research processes. In the interviews, the most frequently cited reason that participants failed to recruit patients was simply that they forgot: recruitment moved down their priorities in the face of other demands, especially if suitable patients were infrequently identified. Frequent prompts from research support staff or the research team were felt important to remind staff to consider recruitment.

The chosen statements revealed that the referral process was an issue. If it was too time consuming, slow, or unclear, participants were less likely to refer (#31, #32, #38). Ineffective communication between the clinical and research teams was also a factor (#35). Several participants reported that they found the referral process easy but then highlighted their reliance on research support staff. Interviewee I522’s view was typical: ‘I found it quite easy really, I mean X [Research Support Practitioner] knows the specific criteria. If she wasn’t there I’m not sure how active us therapists would be in the research, I think if her role wasn’t there I think we’d maybe struggle’*.*

It was also important for the recruitment processes to fit in with clinical processes, particularly the way information was presented and stored. Some participants mainly used electronic notes and information, so for them paper-based ‘paperwork’ was unhelpful. In contrast, other participants described how electronic paperwork was off-putting as they had limited access to computers. This meant documentation and information needed to be provided in a variety of formats to suit different locations.

## Discussion

Although there has been much research about barriers to recruitment and strategies to overcome them [5, 10-13] to the authors’ knowledge, this is the first to explicitly ask clinicians about the factors that influence their decision to recruit to a trial. The most frequently raised factor was clinicians’ perceived role as the patients’ advocate. This led to gate-keeping [15] as they tended to anticipate patients’ reaction to being asked to participate and then were less likely to approach patients whom they felt were unlikely to wish to participate. Donovan et al. [6, 7] have also reported a conflict between clinical and research roles but noted their participants did not connect this to recruitment rates. By using a mixed-methods approach, we have been able to ask both focussed questions and explore the reasoning behind responses, which has revealed the wide-ranging clinical, social or personal contexts in which referral decisions are made and contribute to gate-keeping.

Clinicians’ experience and views of the trial intervention and research design also influenced the decision to refer. Not surprisingly, referral was more likely if clinicians felt a sense of ownership of the research and had a positive view of the intervention being evaluated. Poor previous experience or a low opinion of any aspect of the study made referral less likely, indicating a lack of personal equipoise. Given that clinical experience is an element of evidence-based decision-making [15, 16], personal equipoise is probably a naive expectation, particularly for complex interventions or situations when the trial or control intervention is available in clinical practice. The ethical difficulty of a lack of personal equipoise is overcome by the principal of collective equipoise: where a community of experts agrees there is uncertainty of the benefit of one treatment over another [17, 18]. However, it does not address the gate-keeping effect of individual clinicians’ views. The development of recruitment strategies that enable direct contact with potential participants, such as by letter, phone or patients’ self-referral without going through an intermediary (such as a clinician), may limit or overcome this effect. This would enable potentially eligible patients to make their own decisions about participation. As well as increasing recruitment rates, this would improve equity of access and fulfil the pledges of the NHS Constitution that patients would be informed of ‘research studies in which you may be eligible to participate’ [19, 20].

Like previous authors, our participants highlighted the influence of the organisation as a factor impacting recruitment both positively or negatively [21]. Our participants particularly highlighted how working in an organisationally unstable environment with high staff turnover, reducing resources and increasing workloads meant clinicians often simply forgot about recruitment amongst the, higher priority, clinical and organisational demands they juggled. Furthermore, the participants did not have a formal research role in their job; recruitment was undertaken in addition to their usual roles. This contrasts with much other clinical research where recruitment is usually undertaken by dedicated research support staff/nurses or principal investigators with a formal role and time allocation. Several unmet needs to support rehabilitation research were highlighted. Firstly, research support needs to include all members of the multi-disciplinary team, who should be embedded within clinical teams. Secondly, researchers should recognise that staff turnover means work to train, build and maintain effective working relationships with clinical teams needs to be provided long-term; reliance on clinical staff to ‘cascade’ information and training amongst the clinical team is unlikely to be effective.

Finally, the logistics of the research study impacted on recruitment. Like others, our participants highlighted that quick, simple procedures facilitated recruitment [22] but the variability in the way services operated meant flexible, often bespoke processes that align with existing clinical processes, pathways and other ‘ways of working’ were often required.

### Limitations

Like all qualitative research, the generalisability of the findings of the present study are limited: one cannot assume that participants from other professions, geographical areas or research experiences would raise the same issues. Furthermore, this is the first study to explicitly seek the views of those involved in rehabilitation research, which includes different professionals and recruitment processes to most clinical research. However, the similarities between some of our findings and previous reports [23, 24] suggest that views expressed could be reasonably representative.

We combined ‘traditional’ semi-structured interviews with a card-sorting task and geometric coding, a novel technique to explore patterns and combinations amongst participants’ views. The statements on the cards were chosen from the literature and our experience of recruitment, but they were somewhat arbitrary. Wording the statements differently or raising different issues may produce different results. Nevertheless, this mixed-methods approach produced rich, multi-faceted data that would not be obtained by using either technique in isolation.

## Conclusions

Patient- and professional-related factors were the most frequent influence on clinicians’ recruitment decisions, which often had a gate-keeping effect. Participants tended to anticipate patients’ response to an approach and did not give patients opportunities to participate if they felt the response would be negative, impact on treatment plans or the patient-professional relationship. Managerial responsibility to deal with multiple priorities (in which recruitment fell below clinical and organisational demands) was also an important factor. Speed and simplicity of recruitment processes and a good fit with clinical practices enhanced recruitment.

## Additional file

Additional file 1:
**Participant interview schedule.** (DOC 26 kb)

## Abbreviation

AFOOT: Ankle Foot Orthosis for Stroke
