# Supplementary material for: Health care professionals’ views of the factors influencing the decision to refer patients to a stroke rehabilitation trial
Source: Trials. 2015 Dec 18;16:577. doi: 10.1186/s13063-015-1115-1 (PMC4683768; doi:10.1186/s13063-015-1115-1)
Supplement: Additional file 1: — Participant interview schedule. (DOC 26 kb) [file 13063_2015_1115_MOESM1_ESM.doc]

**Appendix 1.** **Participant Interview Schedule**

1. **If you think of ‘research’ what comes to mind?**

Probes: what words would you use (e.g. stressful; time consuming; fun; stimulating; daunting; exciting; inappropriate, etc)

**2.** **Can you tell me about your experiences of clinical research?**

Probes: Is it part of your current work role? Explain

Is it prevalent in your work place? Explain

Where do you feel that the majority of published clinical evidence comes from?

**3. Can you tell me about any specific involvement in research?**

Probes: Can you think of any particular names of trials?

What sort of things did you become involved in?

How did it come about?

**4. What are the barriers/ things that facilitate becoming involved in research?**

Probes: What factors would be important to you?

For local trials how can you get involved?

**5. Have you got anything specific within your CPD, which might involve research?**

Probes: Perhaps you could tell me both short & long-term aspirations.

**6. When did you first hear about the AFOOT study?**

Probes:

- Who introduced it to you
- Where were you?
- Were there things you didn’t understand?
- What were your immediate thoughts? Explain
- Did you immediately think of patients who might be suitable?
- Were there particular things which changed your thinking about the study? Please explain

**7.** CASE SCENARIOS

**A: Can you think about the last stroke patient you treated.**

Probe: Today, yesterday, end of last week?

**B: Tell me a bit about them**

**C: Talk me through your considerations for including them in AFOOT** *[Make sheet of eligibility criteria available if needed]*

REPEAT IF TIME ALLOWS OR Probe: Are there other things that might have influenced the decision for other patients?

**8. Why do you think that we haven’t had more community clinician referrals?**

Probes: How easy do you think it is to refer to the AFOOT trial?

What things do you need to do before referring?

What would you do to refer a patient?

How accessible do you think the trial is?

**9**. **Do you think that there are some patients who should not be included in clinical trials?**

Probes: certain types of patients; certain types of deficits/problems?

**10. Is there anything else that I have failed to ask you in this interview which you feel is important for me to know?**

**11. Is there anything else that you would like to add?**
